# Flexible multimaterial fibers in modern biomedical applications

**DOI:** 10.1093/nsr/nwae333

**Published:** 2024-09-23

**Authors:** Jongwoon Kim, Xiaoting Jia

**Affiliations:** The Bradley Department of Electrical and Computer Engineering, Virginia Tech, Blacksburg, VA 24060, USA; The Bradley Department of Electrical and Computer Engineering, Virginia Tech, Blacksburg, VA 24060, USA; School of Neuroscience, Virginia Tech, Blacksburg, VA 24060, USA; Department of Materials Science and Engineering, Virginia Tech, Blacksburg, VA 24060, USA

**Keywords:** biomedical fiber, thermal drawing, functional fiber, biomedical devices

## Abstract

Biomedical devices are indispensable in modern healthcare, significantly enhancing patients’ quality of life. Recently, there has been a drastic increase in innovations for the fabrication of biomedical devices. Amongst these fabrication methods, the thermal drawing process has emerged as a versatile and scalable process for the development of advanced biomedical devices. By thermally drawing a macroscopic preform, which is meticulously designed and integrated with functional materials, hundreds of meters of multifunctional fibers are produced. These scalable flexible multifunctional fibers are embedded with functionalities such as electrochemical sensing, drug delivery, light delivery, temperature sensing, chemical sensing, pressure sensing, etc. In this review, we summarize the fabrication method of thermally drawn multifunctional fibers and highlight recent developments in thermally drawn fibers for modern biomedical application, including neural interfacing, chemical sensing, tissue engineering, cancer treatment, soft robotics and smart wearables. Finally, we discuss the existing challenges and future directions of this rapidly growing field.

## Teaser

Thermally drawn multimaterial fibers, with their flexibility, multifunctionality and high biocompatibility, are ideal for implantable and wearable biomedical devices, enhancing treatments, minimizing invasiveness and improving diagnostic precision.

## INTRODUCTION

In the late 1970s, telecommunication companies began to widely use fiber optics in their communication network systems [1,2]. For many years, the fabrication and characterization of silica optical fibers have been modified and refined to enhance the optical properties of fibers for the telecommunication industry [2–5]. On the other hand, functional multimaterial fibers have been developed in recent decades to explore other various modalities, such as electrical, chemical and magnetic modalities, delving into novel microstructure fiber designs and a wide range of materials, including polymers, metals, semiconductors, composites, etc. [6–11]. Similarly to the fabrication process for silica optical fibers, a preform—a macroscale design of the desired functional fiber—is heated inside a furnace and controllably drawn to produce a fiber in a highly scalable way. The functional fiber inherits the proportionally miniaturized cross section of the preform while maintaining all complex functionalities [6–8,10]. Some of these functionalities include piezoelectricity [12–15], triboelectricity [16–20], energy storage [21,22] and optoelectronics [23–28]. Today, fiber technology is implemented in many industries for a variety of applications [6,7,9,10,29–32], such as telecommunications, biomedical devices, environmental sensing, photonics and optoelectronics, defense and aerospace, soft robotics, smart wearables, etc.

Among these applications, biomedical devices are of particular interest, as they play a crucial role in the healthcare system, enabling more effective treatments, less invasive procedures and more precise diagnoses [33–36]. Due to these compelling reasons, the development of new biomedical devices and biomaterials has always been in high demand [36–39]. Thermally drawn multimaterial fibers encompass high flexibility, 1D form factor, multifunctionality and high biocompatibility, making them ideal candidates as implantable and wearable biomedical devices. Indeed, there has been significant progress over the past few years in multimaterial fibers for biomedical applications.

**Figure 1.**
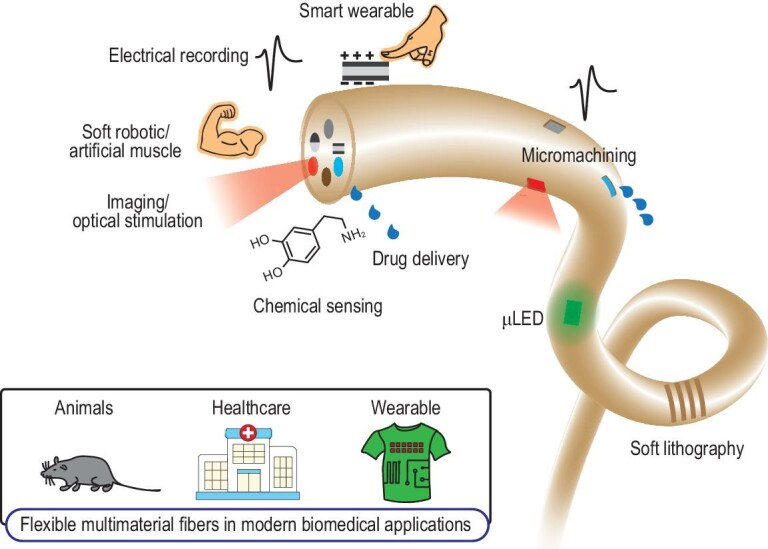
An overview of flexible multifunctional fibers in modern biomedical applications. Through the thermal drawing process, the fibers developed for biomedical fields can record electrochemical activities, deliver light, inject drugs, measure neurotransmitters, actuate muscles and feel pressure. Post-processing along the fiber length, such as surface patterning [97,148,187], bundling [99,188] and microelectronics attachments [100], further increases the functionality and practicality of the fiber technology. Currently, these fibers are utilized and hold significant potential in investigating the organs and central nervous systems (e.g. hearts and brains) of animal models, aiding in diagnosis and treatment in healthcare, and increasing the functionalities of traditional fabrics and textiles.

Here, we review the recent advancement of thermally drawn multimaterial fibers for modern biomedical applications. We first introduce the fabrication method of thermal drawing and the materials embedded in the flexible fibers that enable useful functionalities. Then, we present the biomedical applications of the functional fibers, namely in neural interfaces, tissue engineering, tumor and cancer treatment, programmable soft robotics and smart wearables (Fig. 1). Finally, we discuss the current challenges and future perspectives and directions for the flexible functional fibers.

## FABRICATION METHODS OF THERMAL DRAWING

The very first step for fabricating functional fibers through the thermal drawing process is the design and manufacture of a preform [6,7,9,40]. A preform is a macroscopic structure that has the scaled-up but identical cross-sectional geometry and materials as the final fiber [41]. The cross-sectional dimensions and length of the preform determine the overall length of the fiber [6,10]. The design and composition of the preform are critical, as they define the properties and functionalities of the produced fiber. The preform can include a wide range of functional materials such as thermoplastics, nanocomposites, metals and semiconductors [6,7,9,10,40]. Some of these materials require an additional step involving the extraction of water molecules that are trapped in the materials. This ensures a stable thermal draw without any defects in the resulting fiber from water vapor. The preform is meticulously designed and assembled by utilizing various approaches including traditional machining techniques (milling, drilling and polishing), thin-film rolling, thermal evaporation, extrusion and stacking [28]. Often, the finished preform is wrapped in a sacrificial layer to increase the stability of the draw and mechanically removed or chemically etched after the thermal draw. Lastly, the preform is consolidated in a vacuum oven at the appropriate temperature.

The preform is mounted in a draw tower, where it is softened and pulled into a fiber [6,7,9,10,40]. The draw tower consists of a furnace, a motor to feed the preform, a capstan to pull and draw the fiber, a tension sensor and a 2D laser scanner for measuring the fiber dimensions. A furnace for drawing polymer fibers typically requires three temperature zones: the top zone preheats the preforms, the middle zone softens the preforms and produces the fibers, and the bottom zone cools the fibers [41]. The precise temperatures of the three zones depend on the thermal properties of the preform materials. It is essential that the preform is heated in the furnace and becomes sufficiently viscous to achieve a stable thermal draw. The fiber dimension and length are controlled by precisely regulating the furnace temperature, the preform feeding speed and the fiber drawing speed. The stress built up in the fiber during a thermal draw is computed from the measurements obtained from the tension sensor and the laser scanner. In some cases, fibers are drawn under high stress to neutralize the surface-tension effect of the melted materials in the preform [28,41].

### Materials and functionalities

The thermal drawing process is a versatile platform and accommodates a wide range of materials to enable the fabrication of functional fibers [6,7,10]. Again, the selection and integration of materials in the preform design are critical, as they specifically impact the fiber functionalities. Some noteworthy intrinsic properties of the materials are mechanical, electrical, thermal, optical, chemical and magnetic [42–46]. Functional materials can serve as essential elements in functional fibers, as they possess specific native properties and behaviors that respond to external stimuli in a useful and controlled manner [47]. To give some examples, shape memory alloys can return to their original shape above a certain temperature [48,49]. Thermoelectric materials convert a temperature gradient into electrical voltage [49,50]. Similarly, piezoelectric materials generate an electrical potential in response to mechanical deformation [51,52]. However, thermally drawing a combination of materials and integrating them into a single fiber is not trivial due to the differences in the thermal and mechanical properties of materials.

Here are some general guidelines for successful thermal drawing of a multimaterial fiber. (i) The preform must have at least one material that withstands the drawing stress while deforming controllably, allowing the preform to transform into a fiber in the furnace neck-down region (viscosity <10^7^ poise) [6,41]. This material may be critical if co-drawn with a low-temperature metal, as the material must withstand high stress to counter the Plateau–Rayleigh capillary instability [28,41]. Additional sacrificial layers of the polymer may be employed to stabilize the thermal draw. (ii) Other materials should have lower viscosity than the material in (i) during the thermal draw. If not, materials may break or crack under the high stress [6,28,41]. For example, a high concentration of carbon in a conductive polymer will probably break during a thermal draw due to high viscosity. One way to avoid the break is to increase the temperature such that the conductive polymer is less viscous during the draw, which might lead to a change in the cladding material. We have included some key polymers that are compatible with the thermal drawing process and their glass transition temperatures in Table 1. (iii) Lastly, materials should exhibit similar thermal properties such that there is no breakage or cracks caused during the rapid thermal cooling [6,28,41].

**Table 1.** Polymers that are compatible with the thermal drawing process.

Polymers	Glass transition temperature (*T*_g_, °C)	Reference
Polymethyl methacrylate (PMMA)	102	[189]
Cyclic olefin copolymer (COC)	20–260	[190]
Polycarbonate (PC)	148	[191]
Polyvinylidene fluoride (PVDF)	–35	[192]
Polylactic acid (PLA)	60	[193]
Low-density polyethylene (LDPE)	–100	[194]
High-density polyethylene (HDPE)	–100	[194]
Styrene–ethylene–butylene styrene (SEBS)	–50	[195]
Polyetherimide (PEI)	215	[196]
Polystyrene (PS)	95	[194]
Polycaprolactone (PCL)	–60	[197]

On the other hand, the recently developed convergence drawing process enables the co-drawing of multiple materials while violating some guidelines mentioned above. In the case of convergence drawing [10,53], the preform is fabricated by using empty channels into which materials with a high melting (*T*_m_) temperature (e.g. tungsten microwires and microelectronics) are fed during the thermal drawing. In the neck-down region, the preform is pulled into a fiber and the empty channels collapse onto the high-*T*_m_ material, encapsulating it in place. It is critical that the capstan speed is identical to the feeding speed of the high-*T*_m_ material to avoid a deformed fiber or breakages in the high-*T*_m_ material. This method has broadened the number of possible materials that can be thermally co-drawn, enabling and enhancing functionalities such as superior electrical properties and the embedment of microelectronics.

## BIOMEDICAL APPLICATIONS

The development and refinement of fabrication methods to enhance performance, functionality, biocompatibility and scalability are essential to the creation of new biomedical devices. Some of the common fabrication methods include clean-room microfabrication methods (photolithography and soft lithography) [39,54–56], 3D printing (additive manufacturing) [34,57,58], laser machining [59,60], thermal drawing [6,7] and electrospinning [61–63]. The choice of fabrication methods heavily depends on the materials, geometry and functionalities of the biomedical devices. Currently, the thermal drawing process has proven to be an excellent scalable fabrication platform for neural interfaces, tissue engineering, tumor/cancer treatment, soft robotics and smart wearables. The first generation of multifunctional fibers for biomedical applications have been highlighted and reviewed by several groups [6,7,10,64,65]. These reviews provide a comprehensive evaluation and examination of the fiber-based biomedical devices. Since then, many new flexible multimaterial fibers have been developed for various biomedical subfields, which are presented and discussed in this review.

### Application in neural interfaces

Implantable neural interfaces are important tools for studying neural dynamics and find treatments and cures for neurological disorders and diseases [66–69]. Over the years, tetrodes [70], Utah arrays [71], silicon probes [72,73] and flexible probes [74–77] have been commonly used for the high-quality recording of extracellular activities (single units and local field potential (LFP)) [78–80]. With the recent advancements in fluorescence sensors [81,82] and optogenetics [83–85], the identification, recording and manipulation of different cell types have been widely used via light delivery [86]. This advancement has led into an explosion of innovative bidirectional devices with electrical and optical modalities [87–91]. These devices usually require electrodes with impedances of <2 MΩ, high temporal and intensity-sensitive light delivery, and low flow-rate drug delivery. Amongst many new neural probes, polymer fiber probes, fabricated using the thermal drawing process, are being actively developed and utilized as flexible and multifunctional neural interfaces to study brain circuitry.

**Figure 2.**
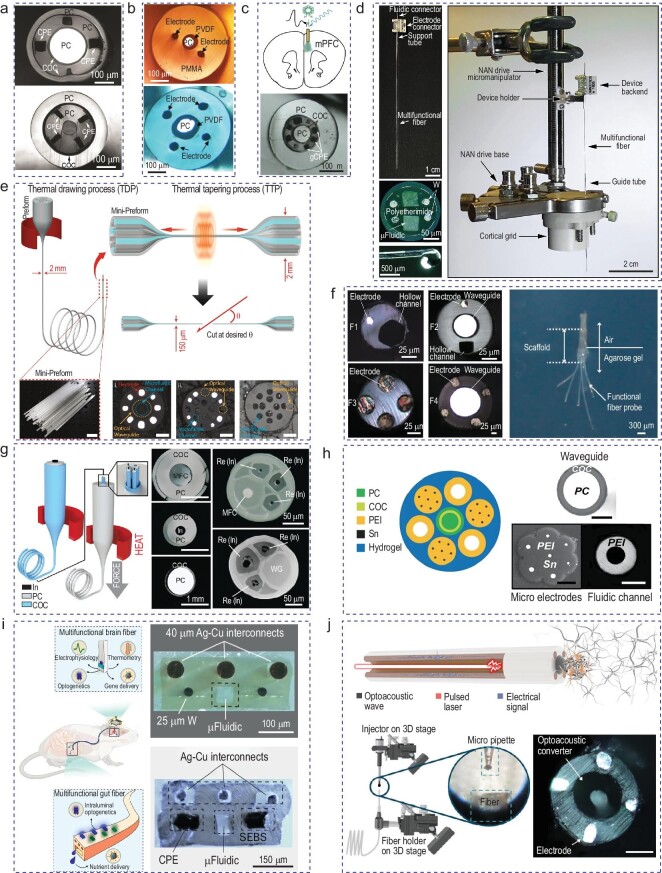
Thermally drawn multifunctional fibers for neural interface. (a) Cross sections of the first realization of neural fiber probes. Adapted with permission from [92]. Copyright 2015 Springer Nature. (b) Cross sections of fiber neural probes with metal microwires using the convergence thermal drawing process. Adapted with permission from [93]. Copyright 2020 John Wiley and Sons. (c) One-step optogenetic fiber probe allows co-localized expression, recording and optogenetics. Adapted with permission from [94]. Copyright 2017 Springer Nature. (d) First implantation of multifunctional fiber neural probe in a non-human primate. Adapted with permission from [95]. Copyright 2023 AAAS. (e) Thermal tapering method to ease the back-end connections of fiber probes. Scale bars: 2 cm (inset in the bottom left corner), 200 μm (the three insets in the bottom right corner). Adapted with permission from [96]. Copyright 2024 Springer Nature. (f) Spatially expandable fiber probes enabling multisite recording and manipulation across different regions. Adapted with permission from [97]. Copyright 2020 Springer Nature. (g) MRI-compatible multifunctional fiber neural probe. Adapted with permission from [98]. Copyright 2021 John Wiley and Sons. (h) Modular integration of fiber probes and hydrogel to increase biocompatibility. Scale bars: 50 μm. Adapted with permission from [99]. Copyright 2021 Springer Nature. (i) Simultaneous interrogation of sensing and delivery of sucrose in the gut while recording from the brain. Adapted with permission from [100]. Copyright 2024 Springer Nature. (j) Optoacoustic fiber probe to stimulate neuronal activity. Scale bar: 100 μm. Adapted with permission from [101]. Copyright 2023 John Wiley and Sons.

The scalable thermal drawing process makes a very appealing platform for the fabrication of neural probes due to its flexibility, multifunctionality, high yield and the inherent geometry of a fiber, which is suitable for implants. Figure 2a shows the first realization of a thermally drawn polymer fiber as a flexible, multifunctional neural interface [92]. In this fiber, conductive polyethylene (CPE) served as the recording electrodes. Polycarbonate (PC) wrapped in cyclic olefine copolymer was utilized as the optical waveguide of the fiber for optogenetic stimulation of neurons. The microfluidic channel enabled focal drug delivery at the tip of the fiber. In Fig. 2b, platinum, gold and copper were drawn with the fiber via the convergence thermal drawing process, enabling lower impedance and therefore an improved signal-to-noise ratio compared with the conductive composites [93]. Using the multifunctional property of the fibers, researchers have also demonstrated one-step optogenetics in the wild-type mouse by infusing viral vectors to express channelrhodopsin 2 in the medial prefrontal cortex by using with the microfluidic channel of the fiber probe, as shown in Fig. 2c [94]. This process removes the extra steps of cannula extraction and probe insertion, and enables co-localized expression, light delivery and electrophysiology recording. Figure 2d presents a flexible electrofluidic neural interface for non-human primates with four electrodes and two microfluidic channels [95]. In this work, Garwood *et al.* locally infused γ-aminobutyric acid into the premotor cortex and putamen of a rhesus macaque while simultaneously recording the electrophysiology. Although they found some changes in the single-unit firing rate and oscillatory structure of the LFP after infusion, they did not observe any consistent changes in behavior.

From this initial generation of fiber neural probes, several innovative designs and/or additional methods have been included to enhance the feature density and reduce tissue damage. Recently, a thermal tapering process was developed to overcome the inherent challenge in back-end connection (Fig. 2e). The tip of the probe is miniaturized to reduce tissue damage while the large back end enables industrial-scale connectorization [96]. By using the tri-modality of the probe, Kim *et al.* found that the local infusion of a cannabinoid receptor agonist is sufficient to disrupt the generation of sharp wave ripples regardless of the Hippocampus Cornu Ammonis 3 inputs. As a result, the thermal drawing process is an excellent platform for the development and fabrication of novel deep-brain neural interfaces in order to study and understand brain circuits. Figure 2f presents a platform that enables fiber probes to interface with the brain in three dimensions via a helical scaffold [97]. The spatially expandable probes allow the simultaneous monitoring and manipulation of neural activities from different regions of the brain by using a single insertion point. By measuring electrophysiology from different regions of the brain in an epileptic mouse model, this probe showed promising application in studying neurological diseases across different regions. In Fig. 2g and a two-step and convergence thermal drawing was revisited for consistent and easier fiber drawing [98]. These neural probes were mounted on a custom-developed microdrive to enable depth-resolved investigation of neural circuits. Furthermore, the fiber probes showed a high compatibility with magnetic resonance imaging (MRI), even with the infusion of an MRI contrast agent through the microfluidic channel. To enhance the biocompatibility and feature density of the fiber neural probes, Park *et al.* devised a hybrid fiber probe by integrating the polymer fibers within a hydrogel matrix [99]. Figure 2h illustrates the fibers that were used in the fabrication of the hybrid probe and the hydrogel bonding techniques. These probes chronically recorded sorted single units over the 6 months that followed implantation.

Apart from increases in feature density and biocompatibility, the introduction of new functionalities has been shown in recent years. By using the multifunctionality of the flexible fibers to its fullest potential, microelectronic fibers were implanted into the ventral tegmental area (VTA) and gut (Fig. 2i) [100]. By coupling these fiber probes to a NeuroStack, wireless optogenetic modulations in the brain and the gut were achieved. Sucrose solution was injected through the microfluidic channel in the gut probe while the spike activity in the VTA neural probe was simultaneously recorded. By utilizing these probes, they discovered that the optogenetic modulation of vagal afferents from intestinal lumen was enough to induce a reward phenotype. Apart from using optogenetics or electrical stimulation to manipulate neural activity, Fig. 2j presents a multifunctional fiber-based optoacoustic emitter probe to excite neurons with acoustic waves [101]. By using a controlled micro-injection process, an optoacoustic coating was deposited at the tip of the optical waveguide, which was fabricated via the thermal drawing process. With nanosecond pulses of a 16.6-uJ laser, a peak-to-peak pressure of 1.0 MPa was measured, which is sufficient to stimulate a neuron. Due to the intrinsic ability of neurons to respond to acoustic waves, no viral transfection is required for this specific modulation. As a result, the thermal drawing process is an excellent platform for the development and fabrication of novel deep-brain neural interfaces in order to study and understand brain circuits.

In the central nervous system, balanced neurochemicals are essential for maintaining a healthy brain function [102,103]. Recent studies have revealed that neurological, neurodegenerative and psychiatric diseases are directly associated with neurochemical abnormalities in the brain [104,105]. However, the presence of high concentrations of interferent molecules forms a formidable obstacle against the ability of current technologies to accurately monitor the low concentrations of targeted neurochemicals [106]. Microdialysis is a conventional FDA-approved sampling technique for monitoring extracellular concentration [107–110]. However, time delays and the concentration diffusion of the targeted neural chemicals are unavoidable due to the inherent complications in the sampling techniques [110]. Over recent years, pH and neurochemical sensing fibers have been developed to record real-time neurochemical fluctuations to avoid the concentration diffusion from microdialysis.

**Figure 3.**
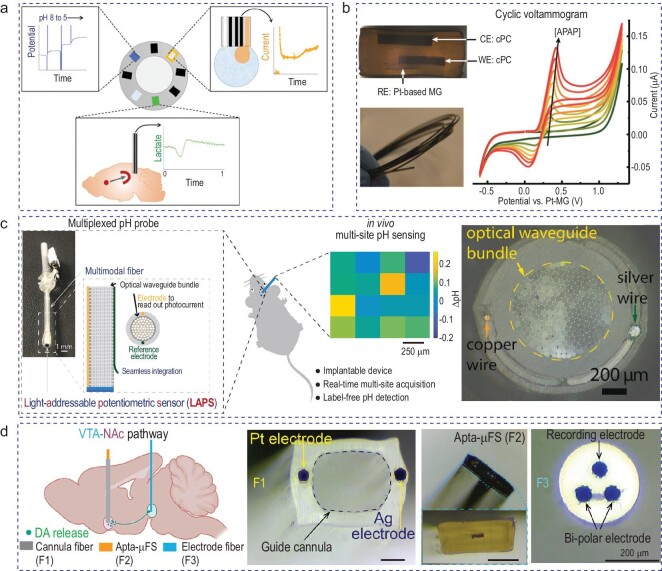
Chemical-sensing fibers developed for neural applications. (a) Fiber electrodes doped with graphite for pH and neurometabolic lactate sensing in the brain. Adapted with permission from [115]. Copyright 2021 American Chemical Society. (b) Fiber sensor that uses cyclic voltammetry and chronoamperometry to quantify paracetamol. Adapted with permission from [116]. Copyright 2021 American Chemical Society. (c) Utilizing a light-addressable potentiometric sensor integrated with fiber probe, spatially resolved and label-free pH sensing was realized. Adapted with permission from [117]. Copyright 2021 Elsevier. (d) Dopamine sensing and electrophysiology recording using aptamers on the fiber sensing tip. Adapted with permission from [118]. Copyright 2023 American Chemical Society.

In-brain pH has been shown to be a critical factor in maintaining normal brain function [105,111–114]. As shown in Fig. 3a, Booth *et al.* developed two designs of a fiber probe with graphite-doped electrodes to monitor pH and neurometabolic lactate [115]. For pH sensors, iridium oxide was layered on the electrodes, which were responsive to pH within the physiological range. For the lactate biosensors, enzyme was layered on top of the platinum black growth on the fiber electrodes. These probes were tested in an *in vivo* mouse model, presenting tools in order to study molecular mechanisms in the brain. Figure 3b presents a three-electrode (conductive polycarbonate and platinum-based metallic glasses) electrochemical fiber sensor [116]. Richard *et al.* used cyclic voltammetry and chronoamperometry to detect and quantify paracetamol—a commonly used anesthetic drug. They successfully conducted cyclic voltammetry by using a portable system for concentrations of between 50 and 300 μM. As shown in Fig. 3c, Guo *et al.* developed a pH fiber probe for spatially resolved and label-free pH sensing by utilizing a light-addressable potentiometric sensor coupled to a flexible multimodal fiber [117]. This probe enabled the monitoring of pH changes of >14 pixels simultaneously with a spatial and temporal resolution of 250 µm and 30 Hz, respectively. In this work, the probes were implanted into the hippocampus of rats and pH changes were recorded over multiple pixels under both physiological and pathological conditions. Figure 3d demonstrates a fiber probe that enables neurochemical sensing with high sensitivity and selectivity [118]. Saizaki *et al.* developed a method to immobilize aptamers on the fiber microsensor. The aptamers were tagged with ferrocene as the electrochemical readout of the binding between the aptamer and the target molecule. In this work, they focused on dopamine sensing and showed a high detection of dopamine selectivity in complex *in vivo* settings. They successfully monitored electrically induced dopamine release, with a practical low detection down to 5 nM and electrophysiology across different brain regions. Overall, the thermal drawing process is a promising fabrication method for neurochemical sensing. However, the neuroscience community would greatly benefit from multifunctional fibers with photometry capability, as photometry is widely used for neurochemical sensing nowadays.

### Application in tissue engineering

Injured neural tissue and pathways often result in life-long discomfort and disabilities [119–122]. A nerve guidance scaffold to promote axonal neural growth can provide a therapeutic solution for severed neural pathways [123–126]. Commercially available guidance scaffolds are often limited by either flexibility, geometry, length or biocompatibility [127]. Recently, new scaffolds have been developed through many different methods such as molding, extrusion, freeze-drying, 3D printing, etc. [125,126,128–132].

**Figure 4.**
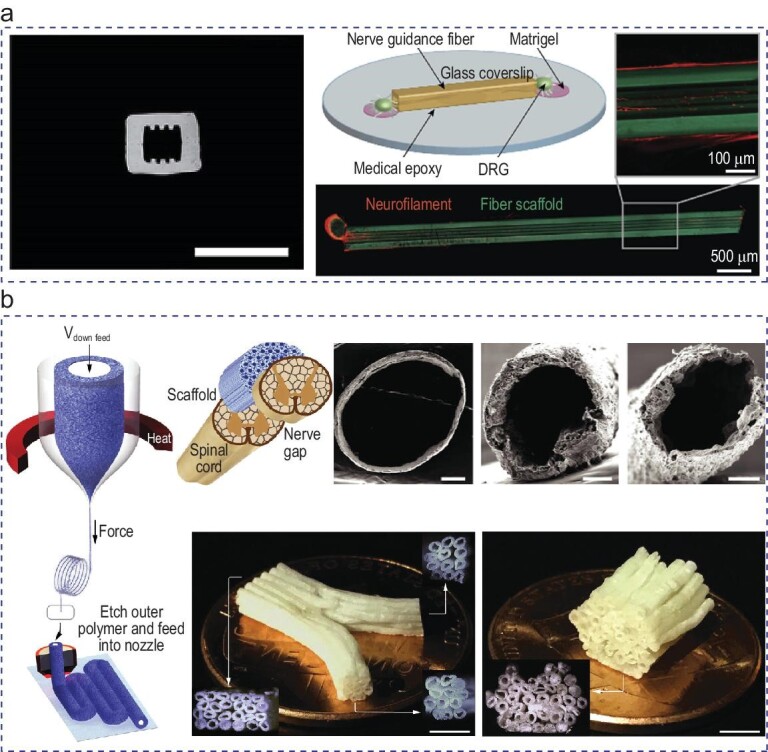
Thermally drawn fibers as a nerve guidance scaffold. (a) Polyetherimide fiber with 50-µm inner diameter and microgrooves facilitates growing neurites. Scale bar: 200 μm. Adapted with permission from [133]. Copyright 2016 Elsevier. (b) Porous fiber scaffolds, utilizing filament surface heating fuse-printing and thermal drawing, enhanced sensory neuron growth. Scale bars: 500, 200 and 100 μm (top, from left to right); scale bars: 2 mm (bottom). Adapted with permission from [129]. Copyright 2019 John Wiley and Sons.

A couple of innovative regenerative scaffolds have been fabricated by using the thermal drawing process, investigating the appropriate geometry and materials for axonal growth. As shown in Fig. 4a, Koppes *et al.* fabricated a polyetherimide nerve guidance scaffold with variation in the geometry and core size (50–200 µm) [133]. The scaffolds with dimensions of >40 and <150 µm were likely to host growing neurites. They found that microgrooves enhanced the neurite growth within the thermally drawn fiber scaffold. Figure 4b shows the regenerative scaffold produced by using fiber drawing combined with salt leaching, which enabled tunable cross sections and porosity [129]. The porous fibers were further arranged into a complex scaffold geometry by utilizing filament surface heating fuse-printing. These porous scaffolds showed enhanced performance in sensory neuron growth compared with non-porous scaffolds with identical materials and geometry. As a result, the scalable thermal drawing process is a promising platform for neural tissue engineering due to its fine control over microscale features and adaptability to a wide range of biocompatible materials.

### Applications in cancer/tumor treatment

Cancer ranks as the second-leading cause of death globally [134,135]. It is characterized primarily by the abnormal ability of tumor cells to proliferate and withstand apoptosis [136], which is essential for their resistance to conventional treatments such as chemotherapy and radiotherapy [137–141]. Traditionally, treatment options have been limited to surgery, radiation, chemotherapy or in combination. However, recent advancements have highlighted the complex pathways involved in the progression of cancer and how they can be targeted for therapy [138,142,143]. This has led to the development of new treatments, including drugs, biological agents and immune therapies, which offer hope for extending the lives of those with metastatic cancer [138].

**Figure 5.**
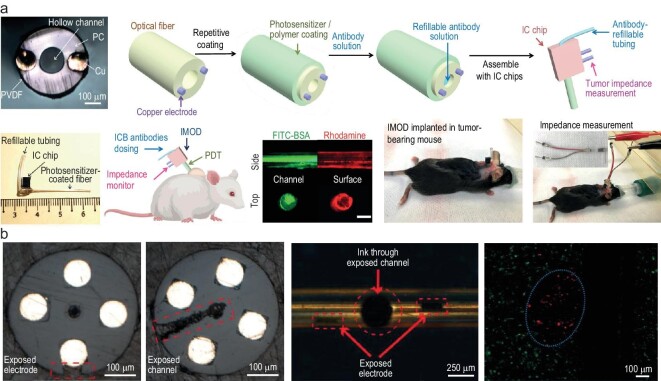
Multifunctional fibers in cancer and tumor treatment. (a) Implantable fiber probe to deliver antibodies and monitor tumor impedance measurements. Scale bar: 200 μm. Adapted with permission from [144]. Copyright 2021 Springer Nature. (b) Fibers with exposed microfluidic channels and electrodes enable microscale electroporation to ablate tumor cells. Adapted with permission from [148]. Copyright 2022 Springer Nature.

Current drug-delivery strategies cannot sustainably supply drugs, monitor therapeutic responses and adjust drug doses over the course of weeks. Chin *et al.* developed an implantable fiber probe to deliver immune checkpoint blockade (ICB) antibodies through the microfluidic channel and monitor tumor impedance measurements by using electrodes over a few weeks (Fig. 5a) [144]. By using the optical waveguide in the device, they also applied photodynamic therapy, which enhanced antitumor immunity and lengthened intratumoral drug retention. They discovered in multiple tumor models that photodynamic therapy with local ICB antibodies delivery cures/delays tumor growth. In addition, irreversible electroporation (IRE) has some key advantages over traditional methods, such as a reduction in treatment time, absence of heat effects, sharp ablation regions and tissue selectivity [133,145–147]. IRE treatment applies pulses of a high-magnitude electric field to increase the permeability of the cell, thereby disturbing the cellular homeostasis and leading to cell death [145,147]. By using thermal drawing process and femtosecond laser micromachining technique, Kim *et al.* developed a flexible fiber-based microscale electroporation device (Fig. 5b) [148] that demonstrated microscale IRE in a 3D collagen scaffold seeded with U251 human glioma cells.

### Application in programmable soft robotics

From prosthetics and endoscopy to minimally invasive surgery, multifunctional soft robotics hold high potential in biomedical applications [35,149–153]. These soft robotics are often capable of navigating through biological environments with high sensitivity through various methods [154,155] such as pneumatics [156], hydraulics [157], shape memory [158] and magnetics [159]. Additionally, they are equipped with sensing and/or actuating modalities that enable functionalities such as imaging, monitoring, optical stimulation and drug delivery [159–162]. Many soft robotics have been developed through fabrication techniques including molding, 3D printing and thin-film electronic fabrication techniques [153,155,163,164]. However, soft robotics are often limited by the materials and geometry that are realizable when using these manufacturing methods.

**Figure 6.**
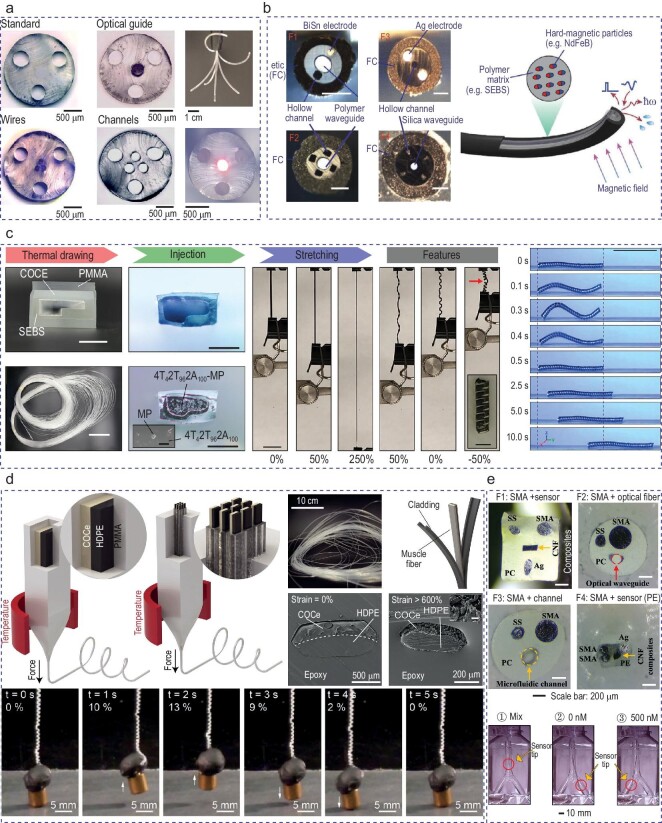
Multifunctional flexible fibers as soft robotics. (a) Tendon-driven technique to accurately move the fiber along with the multifunctionality. Adapted with permission from [152]. Copyright 2022 John Wiley and Sons. (b) Submillimeter fiber robot, which is navigated by an external magnetic field, records and stimulates via electrodes, and delivers light and drugs. Scale bars: 150 μm. Adapted with permission from [165]. Copyright 2023 John Wiley and Sons. (c) Bimorphic elastomeric fiber, fabricated with macroscale strain and magnetization programming, to store cargo, and navigate and release cargo. Scale bars: 10 cm (thermal drawing: top), 1 cm (thermal drawing: bottom), 500 μm (injection: top), 100 μm (injection: bottom), 20 μm (stretching), 1 mm (features) and 1 mm (rightmost). Adapted with permission from [166]. Copyright 2023 John Wiley and Sons. (d) Strain-programmable artificial muscle capable of lifting 650 times its own weight. Adapted with permission from [167]. Copyright 2019 AAAS. (e) Biosensing and light and drug-delivery fiber is steered with shape memory alloys. Adapted with permission from [168]. Copyright 2023 American Chemical Society.

The thermal drawing process is a promising platform for innovating soft robotics in tubular form that are favorable in biomedical applications such as endoscopy and minimally invasive procedures. As shown in Fig. 6a, Leber *et al.* demonstrated thermal drawing as an excellent platform for soft microstructure fiber robotics with complex and dynamic motion navigation with multiple advanced functionalities [152]. The highly accurate motion was achievable by using tendon-driven techniques that were supported by a kinematic model. An optical waveguide, electrical wire and fluidic channels within the fiber robotics enabled advanced functionalities such as 3D scanning, proximity sensing and optical delivery. This robotic fiber shows great potential for minimally invasive surgeries, capable of displacement sensing and imaging, and adapting autonomously to their surroundings.

Recently, researchers have developed soft robotic fibers that can be controlled by using an external magnetic field and can be steered in space. Submillimeter fiber robots, as presented in Fig. 6b, have been developed that can navigate with external magnetic fields, record and stimulate via electrodes, and deliver optical stimulation and pharmacological drugs [165]. By utilizing the thermal drawing process, Zhang *et al.* integrated a fiber with electrical, optical and microfluidic components with a ferromagnetic jacket around the fiber. The robotic fiber was inserted in a Langendorff-perfused mouse heart model, recorded an intracardiac electrogram, monitored bioimpedance and performed pacing. IRE was demonstrated to abolish tumor cells by utilizing the electrodes at the tip and imaging capability through the optical waveguide was illustrated in this study. Three-dimensional soft robotics, as shown in Fig. 6c, are fabricated by using a combination of the thermal drawing process, macroscale strain and magnetization programming [166]. To fabricate a bimorphic elastomeric fiber, cyclic olefin copolymer elastomer (COCe) and styrene–ethylene–butylene–styrene (SEBS) with empty channels were thermally drawn. Then, ferromagnetic neodymium–iron–boron (Nd2Fe14B) alloy particles with a photocurable elastomer was infused into the empty channel of the SEBS. After macroscale lateral strain and magnetization, these robots can be controlled by using unidirectional magnetic fields. Lee *et al.* demonstrated cargo storage, navigation through space and the release of cargo by modulating the unidirectional magnetic field.

An additional way to actuate soft robotics is through changing the temperature. By exploring the differential expansion within polymer bimorph structures, Kanik *et al.* fabricated strain-programmable artificial muscles by using the thermal drawing process [167]. As shown in Fig. 6d, COCe and high-density polyethylene (PE) were thermally drawn with poly(methyl methacrylate) cladding. These fibers were cold drawn at a strain of 50%–1300% to manufacture an actuated spring due to the deformation in the PE. These spring-like actuators withstand >1000% axial deformation and can lift >650 times their own weight with thermal and optical manipulation, highlighting their potential in robotics and prosthetic limb applications. As shown in Fig. 6e, Sato *et al.* developed a steerable fiber sensor consisting of a shape-memory alloy, such as NiTi or NiTiCu, and PC or PE as cladding [168]. A carbon nanofiber composite, optical waveguide and microfluidic channel are embedded into the steerable fiber to enable biosensing, drug delivery and light stimulation. The tip of the fiber was actuated with high spatio-temporal resolution by electrically activating the shape-memory alloy. By additionally using their carbon nano composite, they showed selective detection of adrenaline as low as 100 nM. They targeted a bifurcated vessel model and acquired a localized adrenaline readout by utilizing their fiber sensor, illustrating the thermal drawing process as an outstanding platform for the fabrication of soft and flexible tubular robotics.

### Application in smart wearables

Flexible functional wearables and textiles are emerging as an ideal platform for many biomedical applications such as health monitoring, human–machine interfaces and motion tracking [169–174]. Specifically, smart wearables and textiles can offer useful insights into the user's health condition, which can be utilized for disease prevention, enhanced clinical outcome and improved quality of life [172,175]. Many existing flexible skin-like sensors have been engineered by using thin-film fabrication techniques [39,54,55,172,176]. However, the thermal drawing process has emerged as a powerful and scalable platform for the manufacture of smart fabrics. These smart fibers are weaved into everyday wearables and textiles, providing additional functions such as pressure and temperature sensing [6,7]. Additionally, integration of the fiber through weaving presents certain benefits such as breathability, durability and wear resistance [6,7].

**Figure 7.**
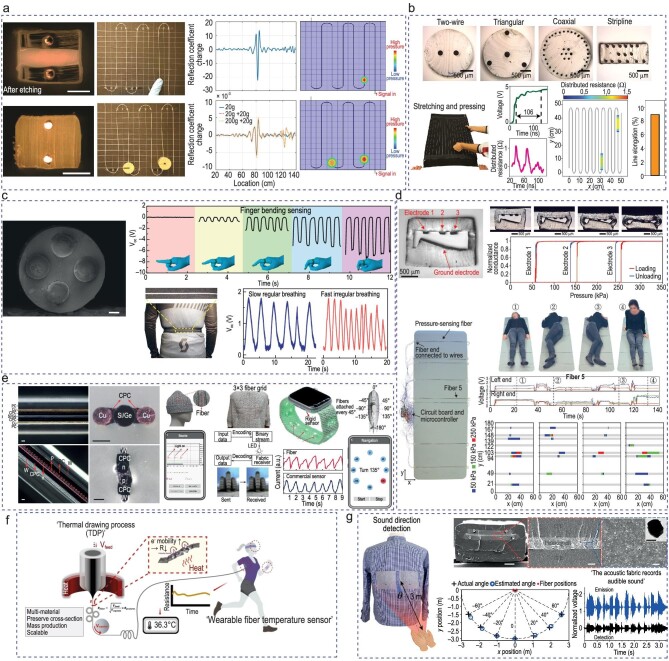
Functional fiber in smart wearables. (a) Using frequency domain reflectometry, pressure points and temperature were measured in the fiber. Adapted with permission from [181]. Scale bars: 200 μm. Copyright 2020 John Wiley and Sons. (b) Elastomeric fiber with liquid metal used time-domain reflectometry to detect pressure point even during deformation. Adapted with permission from [182]. Copyright 2020 Springer Nature. (c) Triboelectric fiber to detect motion of finger flexing and breathing. Scale bar: 200 μm. Adapted with permission from [17]. Copyright 2020 Springer Nature. (d) Electrode contacts in elastomeric fiber measure pressure points on a mat. Adapted with permission from [183]. Copyright 2019 John Wiley and Sons. (e) Optoelectronic fiber that responds to light, enabling light-based communication and photoplethysmography. Scale bars: 50 μm. Adapted with permission from [24]. Copyright 2024 Springer Nature. (f) Temperature-sensing fiber with high sensitivity within the physiological range. Adapted with permission from [185]. Copyright 2023 Springer Nature. (g) Acoustic fiber which can detect sound and operate as a microphone. Scale bars: 20 μm, 2 μm and 200 nm (from left to right). Adapted with permission from [13]. Copyright 2022 Springer Nature.

Pressure sensing enables the detection of mechanical stimulation and provides insights into the compression, elongation, bending and torsion of the fabric [176,177]. In clinical settings, real-time recording of the pressure exerted by the human body on seats, beds and shoes can enable faster diagnosis, and personalized treatment and intervention. Usually, pressure sensing in many functional wearables and textiles is realized by fluctuations in electrical resistance, capacitance, piezoelectricity or triboelectricity [177–180]. As shown in Fig. 7a, Yu *et al.* presented a thermally drawn functional fiber that was capable of measuring pressure or temperature [181]. The fiber consists of thermoplastic, thermoplastic elastomer and metal electrodes. For pressure sensing, they utilized a vector network analyser to perform frequency domain reflectometry, which precisely provided the location of the pressured region. Using a similar set-up, temperature sensing was realized by the changes in the frequency domain reflectometry when the elastomer expanded or shrank due to temperature fluctuations. The reported pressure sensitivity and the temperature sensitivity were 4 kPa and 2°C, respectively. Figure 7b presents a microstructure elastomeric fiber that consists of tens of liquid metals [182]. The pressure points were detected by using time-domain reflectometry. Even under two modes of deformation, stretching and pressing, the detected region of the pressure points agreed with that of the actual pressure points. A triboelectric fiber with elastomer and liquid metal was fabricated by using the thermal drawing process (Fig. 7c) [17]. By using this fiber, a finger-gesture sensor was developed in which the output of the fiber increased as the bending angle increased. This fiber can also be worn around the torso and the triboelectricity due to the elongation and release motion during breathing can be measured through the output of the fiber. Here, the fibers can be woven into deformable machine-washable textiles with electrical outputs of ≤490 V, 175 nC. To simultaneously locate and quantify the pressure points, Leber *et al.* developed an elastomeric fiber with three small CPE sheets on top and a large CPE sheet at an incline on the bottom, as shown in Fig. 7d [183]. With this clever design, the top electrodes came into consecutive contact with the inclined bottom electrode as the pressure on the fiber increased. These fibers could detect pressures at 50, 150 and 250 kPa. Fibers were integrated onto a gymnastic mat for monitoring pressure distribution on a 2D space, providing information on body position, posture and motion. They successfully demonstrated that this set-up could significantly help patients who are prone to pressure ulcers. Wang *et al.* thermally drew a fiber by using high-performing silicon and germanium as the crystalline semiconductor core with glass cladding. The glass cladding was removed through acid etching and the semiconductor fiber was redrawn with thermoplastics and metal wires via convergence thermal drawing (Fig. 7e) [24]. The resultant optoelectronic fiber was woven into other fabric and this enabled a broad scope of applications. Real-time monitoring of photoresponses on a phone was achieved by weaving the fiber into a beanie. They demonstrated a light fidelity-based indoor communication system through integrating the fibers into daily clothing. By using photoplethysmography, heart rate was monitored through weaving the fiber into a watch band. Lastly, they realized an underwater wireless communication system by using the fiber.

Body temperature acts as a vital marker for an individual's health state, indicating potential infections and cardiovascular problems [184]. Figure 7f presents a temperature fiber sensor that was thermally drawn with a polylactic acid-doped reduced graphene oxide core with linear low-density polyethylene cladding and sacrificial polystyrene [185]. The fiber sensor measured the temperature with high sensitivity and fast response within the physiological range. The sensor exhibited adequate sensitivity (–0.285%/°C) within the temperature range of 25–45°C. The durability of the fiber was tested under various levels of humidity, many washing-machine cycles and exposure to household chemicals. They demonstrated the feasibility of this temperature fiber sensor that was woven into clothing and gloves with reliable performance.

Acoustic modality in biomedical applications has advanced in recent decades, ranging from photoacoustic microscopy and drug delivery to therapeutic ultrasound [186]. Acoustic fabrics have promising potential in acoustic communications, acoustic health indicators such as heartbeat and situational acoustic awareness [13]. Yan *et al.* developed an acoustic fiber that can detect audible sound and operate as a sensitive microphone by utilizing the thermal drawing process (Fig. 7g) [13]. The acoustic sensing and actuating are achievable with a piezoelectric active layer of poly(vinylidene fluoride-trifluoroethylene) (P(VDF-TrFE)) that is loaded with barium titanate (BaTiO3) ceramic particles. This piezocomposite layer has a high piezoelectric charge coefficient of 46 pC/N. The performance of the fiber that was woven into a shirt was demonstrated by successful directional detection and acoustic communication. Close contact between the fiber and the chest enables the fiber to record cardiac signals, presenting promising application skin-interfaced stethoscopes.

## FUTURE DIRECTION AND PERSPECTIVE

The thermal drawing process provides a promising approach for the fabrication of functional fibers by integrating various combinations of materials with consistent cross-sectional geometry along the fiber length. The functional fibers open up a wide range of applications in different fields such as telecommunication, biomedical engineering and photonics and optoelectronics. In this review, we presented thermally drawn flexible functional fibers and their use in the fields of neural probes, chemical sensors, tissue scaffolds, cancer treatment devices, soft robotics and smart wearables. Another important consideration for biomedical devices is their reliability for long-term applications. The polymer-fiber-based systems have shown high reliability for long-term use. For example, Canales *et al.* have demonstrated reliable chronic neural recording in mice by using multifunctional fiber-based devices for ≤2 months [92]. Jiang *et al.* also verified the biocompatibility of fiber-based neural probes for ≤2 months [97]. Smart wearables with functional fibers have been shown to be reliable after 100 washing-machine cycles [185]. By leveraging the scalability of the fabrication process, we envision that these functional fiber-based systems will be used in hospitals, enabling better healthcare and patient experience, and in research facilities, pushing the boundaries of fundamental knowledge about diseases and their cures. Below, we outline some ongoing challenges and potential future directions for thermally drawn flexible multifunctional fibers in biomedical application.

### Improvement in fiber design, materials and functionalities

Currently, there are a limited number of materials that are commonly used for the thermal drawing process. The different thermomechanical properties of the materials limit the possible structure and geometry of a fiber. Although there are pre-existing guidelines for material selection, more rigorous modeling and analysis need to be conducted to achieve an optimal fiber design. Engineering of the molecular properties (e.g. high-density and low-density PE) could enable the thermal drawing of materials with unprecedented structures and properties. In addition, further integration of post-processing processes, such as laser micromachining, soft lithography and 3D printing, along the thermally drawn fiber will increase the functionalities and enable 3D interfacing of the fiber. This may also enable integration of other functional materials that are not compatible with the thermal drawing process. Flexibility has always been a significant advantage of thin thermoplastic fibers, yet these fibers are still stiffer than most organs and tissues. Either a reduction in the dimensions or a change in the materials to integrate more seamlessly with biological systems would enhance the biocompatibility of the fibers. Finally, the functionalities of the sensors and actuators should be improved and/or developed. Chemical sensing with high sensitivity and selectivity *in vivo* can lead to new research and studies, developing new treatments and cures in our healthcare. Different modes of stimulation such as electrical, acoustic and magnetic, along with the biochemical sensing can enable strong closed-loop systems.

### Wireless integration of functional fibers

Wireless biomedical devices hold several advantages over wired equivalents. Wireless devices enable patients to move freely, enhancing patient experience and quality of life. Additionally, wireless systems are typically easier to set up without complex wiring and connection. Wired biomedical devices also increase the risk of infection due to the contamination of physical connections, which can act as pathways through which pathogens can enter the body. Some functional fiber devices can allow bulkier back-end-connected electronics, such as smart textiles weaved into a bed mat for pressure mapping. However, many other fiber devices could benefit from a more compact, wireless back-end connectivity. For example, the wireless integration of sweat-sensing fiber weaved onto a T-shirt could allow real-time sensing during exercises. Fully wireless fiber-based neural probes on freely moving mice can enable new social behavior experiments. It is essential to develop wireless power transfer and data transmission systems for fiber devices, which will open up new opportunities for the integration of advanced functional fiber technology into our daily lives.

### Closed-loop feedback control system, in-fiber computation and artificial intelligence

Multiple functionalities can be embedded into a single fiber, enabling bidirectional sensing and actuating. For example, multifunctional fiber neural probes enable extracellular electrophysiology recording while simultaneously manipulating the nearby neural circuits. A closed-loop feedback system could be applied to bidirectional fiber technology, enabling real-time treatment or care. For instance, on-demand drug infusion or electrical pulses with relevant dosage can be administered after the detection or prediction of an epileptic event. Real-time monitoring of a patient's bed pressure map can enable closed-loop control to disperse high pressures at specific locations to prevent pressure ulcers. In-fiber computation of simple signal processing and data storage can greatly aid the commercial use of fibers. These computations should reduce the bulkiness of back-end electronics for closed-loop control. Some of these computations can be as simple as filtering and denoising, and as complex as machine learning (ML) and artificial intelligence (AI). For example, a sweat-sensing fiber, temperature-sensing fiber, pH-sensing fiber and an AI fiber can notify the user or patient with quality information such as dehydration or medication. Embedding fibers with capabilities of ML and AI would unlock new possibilities in smart wearables, soft robotics, disease treatment and beyond. Overall, we envision that these capabilities will enable next-generation health assessments as well as treatments and alert healthcare workers in a timely manner.
